# Complete Genome Sequence of the Proteorhodopsin-Containing Marine Bacterium *Sediminicola* sp. YIK13

**DOI:** 10.1128/genomeA.01635-15

**Published:** 2016-01-28

**Authors:** Yong Min Kwon, Sang-Jin Kim

**Affiliations:** a Marine Biotechnology Research Division, Korea Institute of Ocean Science and Technology, Ansan, Republic of Korea; b Department of Marine Biotechnology, University of Science and Technology, Daejeon, Republic of Korea; c Marine Biodiversity Institute of Korea, Seocheon, Republic of Korea

## Abstract

*Sediminicola* sp. YIK13 is a marine flavobacterium, isolated from tidal flat sediment. Here, we present the first complete genome sequence of this genus, which consists of 3,569,807 bp with 39.4% GC content. This strain contains proteorhodopsin, as well as retinal biosynthesis genes, allowing it to utilize sunlight as an energy source.

## GENOME ANNOUNCEMENT

Proteorhodopsin (PR) is a retinal-binding membrane protein belonging to the largest family of microbial rhodopsins, which function as light-driven proton pumps (1). PR genes are abundant in various ecosystems, particularly within the open ocean and are widely distributed among numerous bacterial groups, including *Gammaproteobacteria*, *Alphaproteobacteria*, and *Flavobacteria* (2–11). However, PR-containing sediment bacteria have not yet been studied. Here, we report the first complete genome for *Sediminicola* sp. YIK13, which was isolated from tidal flat sediment. The light-driven pumping activity of PR in this strain was directly measured using native cell suspensions and reported previously (12).

The strain YIK13 was isolated from a sample of tidal flat sediment collected at Yeongheung Island, Korea (E126°26′, N37°16′) in January 2009. Analysis of the 16S rRNA gene sequence indicates that the strain is a member of the family *Flavobacteriaceae* and shows the highest similarity to *Sediminicola luteus* CNI-3^T^ (99.5%).

Genomic sequencing was performed using PacBio RS II single-molecule real-time (SMRT) sequencing technology (Pacific Biosciences, USA). A 10-kb insert library was constructed and sequenced, yielding >189.9× average genome coverage. *De novo* assembly of the 131,118 reads (677,903,501-bp total) was conducted using the hierarchical genome-assembly process (HGAP) pipeline in SMRT Analysis version 2.2.0 (Pacific Biosciences). Open reading frames were predicted by NCBI Prokaryotic Genomes Annotation Pipeline (PGAAP) version 2.6 (http://www.ncbi.nlm.nih.gov/genome/annotation_prok). The complete genome of *Sediminicola* sp. YIK13 consists of 3,569,807 bp with 39.4% GC content as a circular chromosome without any plasmid DNA. The genome contained 3,113 protein-coding sequences, 9 rRNAs (5S, 16S, and 23S), and 41 tRNAs with a single rRNA operon.

Genomic analysis of YIK13 revealed the presence of a green light-absorbing PR, 242 amino acids in length, with features typical of proton-pumping rhodopsins, including the DTE (corresponding positions of D85, T89, and D96 positions in bacteriorhodopsin) motif, which acts as proton acceptor and donor (2, 13, 14). The *blh* gene encoding a 15,15′-β-carotene dioxygenase was located adjacent to the PR gene, as seen in other PR-containing genomes of *Bacteriodetes* (7). The genome also contains the genes required for β-carotene biosynthesis, including those encoding *crtB* (phytoene synthase), *crtI* (phytoene desaturase), *crtY* (lycopene cyclase), and *crtA* (spheroidene monooxygenase) (15). Furthermore, it also contained a putative transcriptional regulator (MerR family) in front of the *crtI*, *dxs* (1-deoxy-d-xylulose-5-phosphate synthase), and *idi* (isopentenyl pyrophosphate d-isomerase) genes involved in FPP (farnesyl diphosphate) and IPP (isopentenyl diphosphate) precursor synthesis. Similar to other PR-containing genomes of *Bacteriodetes* (7), this genome also contained the *crtZ* (β-carotene hydroxylase) gene involved in the synthesis of zeaxanthin (a carotenoid pigment). This sequence may provide further information necessary to understand the physiological and ecological functions of PR-mediated light utilization in sediment-dwelling microorganisms.

### Nucleotide sequence accession numbers.

The complete genome sequence of *Sediminicola* sp. YIK13 has been deposited at DDBJ/EMBL/GenBank under the accession number CP010535. The partial sequence of the 16S rRNA gene of *Sediminicola* sp. YIK13 has been deposited into the GenBank database under the accession number JX312334.

## References

[1] Spudich JL, Yang C-S, Jung K-H, Spudich EN 2000 Retinylidene proteins: structures and functions from archaea to humans. Annu Rev Cell Dev Biol 16:365–392. doi:10.1146/annurev.cellbio.16.1.365. 11031241

[2] Béjà O, Aravind L, Koonin EV, Suzuki MT, Hadd A, Nguyen LP, Jovanovich SB, Gates CM, Feldman RA, Spudich JL, Spudich EN, DeLong EF 2000 Bacterial rhodopsin: evidence for a new type of phototrophy in the sea. Science 289:1902–1906. doi:10.1126/science.289.5486.1902. 10988064

[3] Béjà O, Spudich EN, Spudich JL, Leclerc M, DeLong EF 2001 Proteorhodopsin phototrophy in the ocean. Nature 411:786–789. doi:10.1038/35081051. 11459054

[4] Venter JC, Remington K, Heidelberg JF, Halpern AL, Rusch D, Eisen JA, Wu D, Paulsen I, Nelson KE, Nelson W, Fouts DE, Levy S, Knap AH, Lomas MW, Nealson K, White O, Peterson J, Hoffman J, Parsons R, Baden-Tillson H, Pfannkoch C, Rogers YH, Smith HO 2004 Environmental genome shotgun sequencing of the Sargasso Sea. Science 304:66–74. doi:10.1126/science.1093857. 15001713

[5] Sabehi G, Béjà O, Suzuki MT, Preston CM, DeLong EF 2004 Different SAR86 subgroups harbour divergent proteorhodopsins. Environ Microbiol 6:903–910. doi:10.1111/j.1462-2920.2004.00676.x. 15305915

[6] Giovannoni SJ, Bibbs L, Cho J, Stapels MD, Desiderio R, Vergin KL, Rappé MS, Laney S, Wilhelm LJ, Tripp HJ, Mathur EJ, Barofsky DF 2005 Proteorhodopsin in the ubiquitous marine bacterium SAR11. Nature 438:82–85. doi:10.1038/nature04032. 16267553

[7] Gómez-Consarnau L, González JM, Coll-Lladó M, Gourdon P, Pascher T, Neutze R, Pedrós-Alió C, Pinhassi J 2007 Light stimulates growth of proteorhodopsin-containing marine *Flavobacteria*. Nature 445:210–213. doi:10.1038/nature05381. 17215843

[8] Atamna-Ismaeel N, Sabehi G, Sharon I, Witzel K, Labrenz M, Jürgens K, Barkay T, Stomp M, Huisman J, Béjà O 2008 Widespread distribution of proteorhodopsins in freshwater and brackish ecosystems. ISME J 2:656–662. doi:10.1038/ismej.2008.27. 18369329

[9] Campbell BJ, Waidner LA, Cottrell MT, Kirchman DL 2008 Abundant proteorhodopsin genes in the North Atlantic Ocean. Environ Microbiol 10:99–109. doi:10.1111/j.1462-2920.2007.01436.x. 18211270

[10] Koh EY, Atamna-Ismaeel N, Martin A, Cowie ROM, Béjà O, Davy SK, Maas EW, Ryan KG 2010 Proteorhodopsin-bearing bacteria in Antarctic sea ice. Appl Environ Microbiol 76:5918–5925. doi:10.1128/AEM.00562-10. 20601510 PMC2935036

[11] Riedel T, Gómez-Consarnau L, Tomasch J, Martin M, Jarek M, González JM, Spring S, Rohlfs M, Brinkhoff T, Cypionka H, Göker M, Fiebig A, Klein J, Goesmann A, Fuhrman JA, Wagner-Döbler I 2013 Genomics and physiology of a marine flavobacterium encoding a proteorhodopsin and a xanthorhodopsin-like protein. PLoS One 8:e57487. doi:10.1371/journal.pone.0057487. 23526944 PMC3587595

[12] Kwon YM, Kim S-Y, Jung K-H, Kim S-J 13 12 2015 Diversity and functional analysis of light-driven pumping rhodopsins in marine *Flavobacteria*. MicrobiologyOpen. [Epub ahead of print.] doi:10.1002/mbo3.321. PMC4831467 26663527

[13] Man D, Wang W, Sabehi G, Aravind L, Post AF, Massana R, Spudich EN, Spudich JL, Béjà O 2003 Diversification and spectral tuning in marine proteorhodopsins. EMBO J 22:1725–1731. doi:10.1093/emboj/cdg183. 12682005 PMC154475

[14] Fuhrman JA, Schwalbach MS, Stingl U 2008 Proteorhodopsins: an array of physiological roles? Nat Rev Microbiol 6:488–494. doi:10.1038/nrmicro1893. 18475306

[15] Teramoto M, Takaichi S, Inomata Y, Ikenaga H, Misawa N 2003 Structural and functional analysis of a lycopene β-monocyclase gene isolated from a unique marine bacterium that produces myxol. FEBS Lett 545:120–126. doi:10.1016/S0014-5793(03)00513-1. 12804761

